# The Role of Mitochondria in Mediation of Skeletal Muscle Repair

**DOI:** 10.3390/muscles2020011

**Published:** 2023-03-24

**Authors:** Stephen E. Alway, Hector G. Paez, Christopher R. Pitzer

**Affiliations:** 1 Laboratory of Muscle Biology and Sarcopenia, Department of Physical Therapy, College of Health Professions, University of Tennessee Health Science Center, Memphis, TN 38163, USA; 2 Department of Physiology, College of Medicine, University of Tennessee Health Science Center, Memphis, TN 38163, USA; 3 Center for Muscle, Metabolism and Neuropathology, Division of Regenerative and Rehabilitation Sciences, College of Health Professions, University of Tennessee Health Science Center, Memphis, TN 38163, USA; 4 The Tennessee Institute of Regenerative Medicine, 910 Madison Avenue, Memphis, TN 38163, USA; 5 Integrated Biomedical Sciences Graduate Program, College of Graduate Health Sciences, University of Tennessee Health Science Center, Memphis, TN 38163, USA

**Keywords:** mitochondria, mitophagy, satellite cells, muscle stem cells, metabolism, mitophagy, apoptosis, muscle regeneration, cell proliferation, stem cell differentiation

## Abstract

Musculoskeletal health is directly linked to independence and longevity, but disease and aging impairs muscle mass and health. Complete repair after a pathological or physiological muscle injury is critical for maintaining muscle function, yet muscle repair is compromised after disuse, or in conditions such as metabolic diseases, cancer, and aging. Regeneration of damaged tissue is critically dependent upon achieving the optimal function of satellite cells (muscle stem cells, MSCs). MSC remodeling in muscle repair is highly dependent upon its microenvironment, and metabolic health of MSCs, which is dependent on the functional capacity of their mitochondria. Muscle repair is energy demanding and mitochondria provide the primary source for energy production during regeneration. However, disease and aging induce mitochondrial dysfunction, which limits energy production during muscle regeneration. Nevertheless, the role of mitochondria in muscle repair likely extends beyond the production of ATP and mitochondria could provide potentially important regulatory signaling to MSCs during repair from injury. The scope of current research in muscle regeneration extends from molecules to exosomes, largely with the goal of understanding ways to improve MSC function. This review focuses on the role of mitochondria in skeletal muscle myogenesis/regeneration and repair. A therapeutic strategy for improving muscle mitochondrial number and health will be discussed as a means for enhancing muscle regeneration. Highlights: (a). Mitochondrial dysfunction limits muscle regeneration; (b). Muscle stem cell (MSC) function can be modulated by mitochondria; (c). Enhancing mitochondria in MSCs may provide a strategy for improving muscle regeneration after an injury.

## 1. Introduction

Skeletal muscle accounts for ~50% of the lean body mass in a young adult. As one of the largest organs in the body, skeletal muscle has a primary functional role in locomotion and posture, but it is also important in the regulation of exercise, temperature, protein storage, and systemic metabolism [1].

Muscle atrophy occurs through disuse [2,3], prolonged bedrest [4,5], aging [6,7,8,9], cancer cachexia [10,11,12], nerve injury [13], diabetes [14,15], stroke [16,17], and muscle myopathies [18,19,20]. Muscle atrophy and loss increases the prevalence of falls [21] and hospitalizations, reduces the quality of life [22], lowers independence, shortens life span [23,24,25] and increases the risk for metabolic diseases [26], especially in the elderly.

Skeletal muscle maintenance depends on proper proteostasis [6] that supports ongoing repair, regeneration, and growth, which is mediated by muscle stem cells/satellite cells (MSC) [27,28,29]. Repair of skeletal muscle is dependent upon precisely regulated signaling events involving the activation and differentiation of MSCs. The proliferation of MSCs is necessary to replace the quiescent cell pool that will commit to differentiation and therefore be removed from this pool [30,31]. Muscle repair following pathological or most physiological injuries or surgery is critically dependent upon the adequate proliferation of the Pax7 positive population of adult MSCs (i.e., satellite cells), which provides a pool of stem cells for muscle repair. However, various pathologies including muscle aging, atrophy, and unloading/disuse [6,32,33,34,35,36,37,38,39] negatively impact MSC function [40] and reduce the ability of MSCs to regulate muscle regeneration. Thus, assessments of the mechanisms that optimize MSC proliferation and differentiation are critical for establishing strategies to improve neuromuscular function in response to a pathological or physiological injury.

Mitochondrial dysfunction has a role in each of these conditions that leads to muscle atrophy. The relationships between mitochondria quality and muscle mass are not surprising since the maintenance of skeletal muscle mass and function requires substantial energy, which is supplied by the muscle mitochondria. Muscle mitochondria comprise 3–6% of the muscle fiber volume in humans [41,42,43], but mitochondria are not static structures. The quantity of mitochondria can adapt to activity levels, and it varies with muscle fiber type [42]. While mitochondrial abundance improves aerobic capacity and resistance to fatigue [44], fatigue resistance to isometric exercise is not dependent upon fiber abundance of mitochondria [43]. In addition to ATP production [45], mitochondria function has both established and emerging roles in multiple processes in skeletal muscle, including apoptosis [46], autophagy [47,48,49], ion homeostasis [50,51,52], the production of reactive oxygen species (ROS) [2,53,54,55], the control of muscle mass [54,56,57,58] and protein synthesis (Reviewed in [45,46]). Our published data and the work of others implicate dysfunctional mitochondria and diminished mitochondrial turnover as potential mediators of reduced MSC and neuromuscular function [59,60,61,62,63,64,65,66]. Therefore, understanding the mechanisms that maintain the integrity of mitochondrial structure and function is important for overall muscle health and regeneration following an injury.

## 2. Muscle Injury and Regeneration

**Figure 1:**
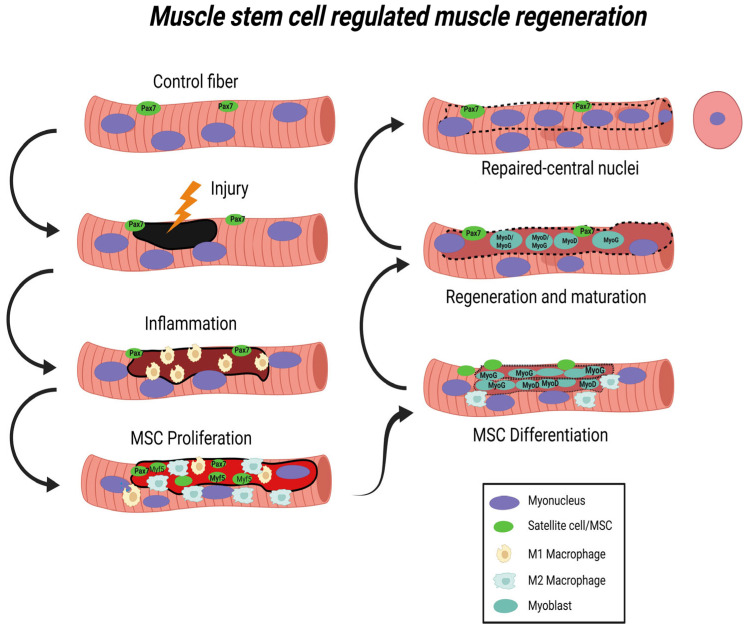
Muscle stem cell regulated muscle regeneration. Muscle regeneration after an injury occurs through a set of programmed events. This includes tissue degradation and inflammation with M1 macrophages. Activation of macrophages in turn activates quiescent muscle stem cells (MSC, satellite cells). The MSCs increase Pax7 abundance and Pax7 acetylation, and MSCs proliferate. Most activated MSCs are Myf5 positive. MSCs reduce Pax7 acetylation, withdrawal from the cell cycle, then differentiate into myoblasts while expressing MyoD and/or Myogenin. The myoblasts migrate into the injured muscle to fuse with other myoblasts to form myotubes, or fuse with the injured muscle cells to complete skeletal muscle repair. This will result in new muscle fibers, or forming a new fiber section that can fuse with undamaged fibers. Some myoblasts fuse to form myotubes. Regenerative fibers have central nuclei (still seen at 21–28 days after an injury) until full remodeling of the fiber occurs, when the nuclei move to a peripheral location. M1, inflammatory macrophage; M2, non-inflammatory macrophage; Pax7, paired-box 7; MyoD, myoblast determination protein 1; MyoG, myogenin; Myf5, myogenic factor 5. Other transcription factors involved in myogenesis are not displayed on this figure.

Skeletal muscle can be injured physiologically by overuse or exercise, or pathologically through military combat, automobile or industrial accidents, sports injuries, or exposure to chemicals, some viruses, or other toxins. Skeletal muscle undergoes repair after physiological or pathological injury through progressive stages that include: degradation of the injured tissue, inflammation to increase removal of damaged tissue and localization of chemosignals for activating MSCs. The regeneration of skeletal muscle proteins typically requires the activation of the MSC/satellite cell dependent system, in which MSCs commit to a myoblast lineage then differentiate by fusing into myotubes to form new muscle fibers or replace portions of damaged or missing fibers. The remodeling and anabolic growth of skeletal muscle then occurs [67,68,69,70,71] to complete the skeletal muscle repair process (Figure 1).

Muscle injury can be induced by many different conditions. Experimentally, pathological damage can be induced and studied in models that include the injection of one of a variety of cobra snake venoms that induces wide-spread degeneration (e.g., notexin, cardiotoxin) [59,72,73,74,75,76,77] which lyses cell cytoplasm but maintains an intact basal lamina as used in our lab [59]. Other methods of injury include the injection of a chemical that causes widespread muscle necrosis (e.g., bupivacaine, BaCl_2_) [78,79,80,81,82]; laceration [83,84] to damage tissue cytoplasm and membranes; insect stings that cause muscle protein disassembly and rhabdomyolysis [85,86,87]; physical interventions such as freezing to induce full tissue and membrane destruction [88,89,90], volumetric muscle loss [77,91,92,93,94,95], laceration [96,97,98,99,100], burn injury [101,102,103,104], muscle crush [105,106,107,108,109,110,111]; or physiological injuries such as downhill running and eccentric loading [112,113,114,115,116,117,118,119,120], overuse, or stretch-shortening injury [113,121,122,123,124,125,126,127]. Although the time course and outcomes of the various injuries differ because in part the injuries to the muscles are not identical by these approaches, muscle will attempt a programmed cycle of muscle repair. The overall pattern is (a) an initial course of inflammation and activation of macrophages, (b) degeneration of the injured tissue, (c) myogenesis, and (d) maturation/remodeling [67,72].

### 2.1. Inflammatory Response to Muscle Injury

The first phase of muscle repair begins with an inflammatory response that is initiated with an almost immediate infiltration of neutrophils. Neutrophils begin clearing damaged organelles and proteins via phagocytosis. They also release chemotactic molecules that provide a homing type of signaling for macrophages to migrate to the site of the injury [128,129]. Macrophages then assume the majority of the task of removing damaged and degraded skeletal muscle proteins, including membranes and organelles [130]. The immune response to injury is an important trigger to help regulate MSC-induced repair [131,132,133,134] and loss of function in the immune system such as in aging, which diminishes the regenerative response to muscle injury [131].

Reactive oxygen and nitrogen species promote an environment of oxidative and nitrosative stress, stimulating inflammation after an injury [135]. Pro-inflammatory phagocytic M1 macrophages are initially activated and are responsible for the efficient removal of damaged tissue [136,137]. After the initial clearance of damaged proteins, there is a transition to the alternative anti-inflammatory non-phagocytic M2 macrophage (Figure 1). This M1 to M2 transition is crucial for the second phase of the regeneration process which then leads to the rebuilding process, or myogenesis [72]. Furthermore, macrophages are important for remodeling the skeletal muscle extracellular matrix (ECM), which provides molecular signaling for muscle growth [138].

### 2.2. Myogenic Responses to Muscle Injury

Macrophages provide cytokines and growth factor signals [139] that activate quiescent (i.e., a reversible G_0_-like state) mononucleated muscle stem cells/satellite cells (MSCs), which are critical for muscle repair following an injury [73,140]. MSCs are also called satellite cells based on their anatomical position [30,141,142]. Satellite cells/MSCs lay outside of the muscle sarcolemma but inside the basal lamina where they are quiescent [143]. After activation, MSCs proliferate [143,144,145] to create a larger pool of MSCs, which is a critical process for initiating muscle regeneration [28,146]. However, after dividing, some MSCs remain outside of the fibers to act as a reservoir of cells for a subsequent insult. The remainder of the dividing MSCs transition into committed myoblasts that migrate inside the damaged cells (fuse with the damaged fiber) [145,147,148] to provide transcriptional support for muscle regeneration [149,150,151]. While increased MSC proliferation can improve muscle regeneration after an injury, a precise balance must be made between optimal proliferation and differentiation of MSCs for complete regeneration [68,152]. Myoblasts may also secrete extracellular vesicles (exosomes) that help to promote muscle regeneration [153]. The repaired fiber has an MSC-derived central nucleus, which is a hallmark phenotype of a fiber that has been injured and has undergone some level of repair requiring MSCs [59,73,80]. Thus, MSCs proliferate, migrate, differentiate, and they can fuse to form de novo skeletal muscle fibers [154,155,156,157] or fuse with the damaged muscle fibers to support regeneration and repair [158]. The extracellular matrix (ECM) also undergoes remodeling during muscle regeneration [159]. This includes removal of ECM that may have been injured along with the fibers, then, the MSC regulated deposition of de novo ECM proteins [80,160]. Often there is an increase in collagen and other ECM proteins early in the regeneration phases, followed by remodeling and a reduction in the overall ECM protein content later. ECM remodeling is critical to anchor the new contractile elements with the muscle membranes so that contractile forces are transmitted properly for normal muscle shortening and function [80,161,162,163,164]. Nevertheless, excessive ECM deposition impairs MSC function [165] and can lead to fibrosis.

## 3. Mitochondrial Regulation of Regeneration after Muscle Injury

**Figure 2:**
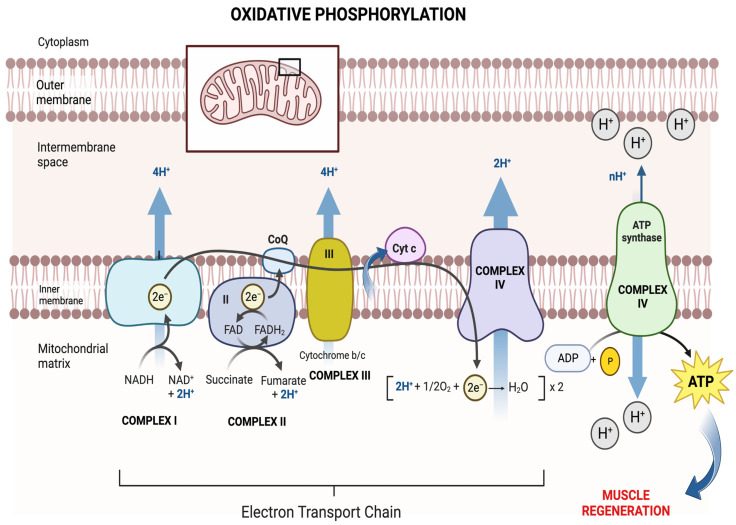
Schematic illustration of the Oxidative Phosphorylation (OXPHOS) complexes. OXPHOS occurs in the inner mitochondrial membrane. Electrons are transferred from NADH or FADH to molecular oxygen which is the terminal electron acceptor. Large transmembrane protein complexes (Complexes I–IV) and the smaller mobile electron carriers make up the structures in the electron transport chain. Complexes I, III and IV generate an electrochemical proton gradient across the membrane (shown as light blue arrows). ATP synthase uses the electron gradient to produce ATP for cellular uses including supplying energy for muscle regeneration.

Mitochondrial function has both established and emerging roles in multiple muscle processes, including adenosine triphosphate (ATP) production through oxidative phosphorylation (OXPHOS, Figure 2) via the mitochondrial electron transport chain (ETC). The high metabolic demand for ATP for myogenesis and the regeneration of skeletal muscle after an injury is achieved via mitochondrial OXPHOS. If mitochondrial health is compromised and the mitochondrial membrane potential (ΔΨ_m_) is lost, mitochondrial ATP generation through OXPHOS is also reduced, and this limits the extent and/or rate of muscle repair.

Mitochondria are critical to proper muscle repair, and mitochondrial dysfunction has been linked to reduced MSC function during repair in aging [166]. In fact, the absence of mitochondrial remodeling has been reported to reduce the differentiation capacity of cultured myoblasts [167,168]. This suggests that mitochondria may not be as critical for proliferation as they are for the processes involved in myoblast differentiation and migration to the injured muscle fibers for participation in cellular repair.

Regeneration of mature myofibers after an injury may require an excess of 21–28 days in healthy young adult rodents, depending on the type of pathological or physiological injury, and the level of damage and the extent of tissue replacement that is needed [59,67,97,132]. The process whereby MSCs/satellite cells proliferate, migrate into the damaged fibers, or fuse to form myotubes to replace damaged fibers is an energy demanding process and requires the remodeling of mitochondria. Inadequate removal of damaged mitochondria, including impaired mitochondrial autophagy (mitophagy), lowers the differentiation capacity of myoblasts in vivo [168,169,170] and reduces the ability of skeletal muscle to fully regenerate [44,171]. Consistent with this idea, reduced mitochondrial biogenesis via the suppression of Peroxisome proliferator-activated receptor-gamma coactivator-1 alpha (PGC1α) is an underlying cause of poor differentiation and maturation/myogenesis of muscle regeneration in atrophic diseases such as Facioscapulohumeral muscular dystrophy (FSHD) [172] and cancer cachexia [173]. PGC1 isoforms have multiple roles. PGC1α is considered a master gene for regulating mitochondrial biogenesis, but it also has several target genes including those implicated in mitochondrial biogenesis (NRF-1, NRF-2), angiogenesis (VEGF-A), and muscle hypertrophy (myostatin/GDF8). PGC1α is also implicated in the phosphorylation of AMPK and MAPK p38 [174].

### 3.1. Mitochondria-Derived Signaling Pathways Controlling Inflammation

#### 3.1.1. Mitochondrial Dynamics

Proinflammatory cytokines including TNFα, IL-1β, IFNβ1 and IL-6 are elevated in sarcopenic muscle and contribute to muscle atrophy and wasting in many conditions such as cancer cachexia. There appears to be a link between mitochondria and inflammation responses in muscle, but it is less clear if inflammation is a cause or a result of aberrant signaling at least in part through mitochondria. Part of the argument that supports a causal role for mitochondria in inflammation stems from observations that a proper balance between mitochondria fission and fusion is required to optimize mitochondrial health and metabolism.

Mitochondrial morphology for fission and fusion induces a distinct inflammatory signature, caused by differential activation of DNA sensors TLR9 or cGAS. Mitofusions 1 and 2 (MFN1/2), along with OPA1, are responsible for mitochondrial regulation of mitochondrial fusion, while DRP1, FIS1, MFF, and MID49/51 promote mitochondrial fission; however, DRP1 appears indispensable for fission [175]. A knockout of MFN1 or MFN2 in multiple tissues including muscle results in growth defects, tissue atrophy, accelerated aging, and systemic inflammation [54,176,177,178]. Interestingly, while mitochondrial dysfunction plays many roles in regulating muscle atrophy, the mitochondrial membrane potential (Δψm) as well as mitochondrial superoxide (mtROS) production, mitochondrial oxygen consumption rates and mitochondrial abundance were reported to not correlate well to the inflammatory profile that occurs upon mitochondrial fragmentation [175,179]. This suggests that fission of mitochondria that leads to fragmentation may not initiate inflammation per se, but regulation of the inflammation pathway, at least through TLR9, does have a role in shaping the inflammatory signature in skeletal muscle. Indeed, other work indicates that skeletal muscle mitochondrial fragmentation promotes TLR9-dependent inflammation [175], muscle atrophy, reduces muscle function, and enhances IL-6 response, which is also an inflammatory cytokine that induces more atrophy [54,175,176,180,181,182,183]. TLR9 is clearly involved in the inflammatory process because TLR9-related inflammation is reduced with anti-inflammatory therapeutics [184,185]. Together, the data suggest that mitochondrial dynamics play an important role in preventing excessive inflammatory responses, which precede the development of muscle atrophy and impaired muscle regeneration in response to an injury. What is also clear is that this area will require additional work to more fully understand the connections of mitochondria remodeling and, particularly, fragmentation and inflammation in skeletal muscle during the onset of muscle injury and the role of mitochondrial regulated inflammation on muscle repair.

#### 3.1.2. Mitophagy and Inflammation

Part of the explanation that mitochondrial dynamics and morphology appear to be connected to inflammation may be through links to mitochondrial specific autophagy, (i.e., mitophagy) [6,176,180,186]; abnormal mitophagy has been in turn thought to activate inflammatory responses [175,179,187]. These conclusions are rooted in observations that mitochondrial stress can lead to the release of damage-associated molecular patterns (DAMPs) to the circulation. Mitochondria-derived DAMPs (mtDAMPs) such as cell-free mitochondrial DNA (mtDNA), mitochondrial transcription factor A (TFAM), and cardiolipin have been linked to chronic inflammation in aging and degenerative diseases [188,189] that activate an immune response. Mitophagy may regulate inflammation [177,190,191,192], at least in part, through nucleotide-binding oligomerization domain-like receptor protein 3 (NLRP3) [193,194,195].

Mitophagy occurs as a result of mitochondrial dysfunction due to a loss of the mitochondrial membrane potential (Δψm) [49]. This triggers a cascade of events that culminate in an autophagic clearance of damaged mitochondria [45]. This clearance of dysfunctional mitochondria is important for maintaining muscle health by moderating proteostasis in aging [6,49]. Normally, the serine/threonine kinase PTEN induced kinase 1 (Pink1) is sequestered within the mitochondrion [196] and its degradation depends on intact Δψm [197]. When mitochondria become damaged (e.g., aging, metabolic diseases, high ROS levels, muscle injury, etc.) and the membrane becomes depolarized, Pink1 is stabilized and accumulates in the outer mitochondrial membrane (OMM) [196,197,198] where it recruits the ubiquitin ligase Parkin. Parkin is cytosolic and inactive under normal conditions in a healthy non-pathological environment [197]. Upon mitochondrial localization, Parkin is activated and ubiquitinates OMM proteins with polyubiquitin chains [6,49,199].

The link between mitophagy and inflammation appears to be very strong. Indeed, the loss of Parkin, an E3 ubiquitin ligase, and Pink1, a ubiquitin kinase—two important proteins that identify damaged mitochondria and trigger mitophagy—results in increased serum levels of proinflammatory cytokines IL-6, IFNβ1, TNFα, IL-1β, CCL2, IL-12(p70), IL-13, IL-17, CXCL1, and CCL4 in mice that were both 20 and 40 weeks of age [187]. Mitophagy may mitigate inflammation, and there is a strong inflammatory phenotype in both Parkin^-/-^ and Pink1^-/-^ mice in non-muscle cells [200,201] and in muscle following exhaustive exercise [187].

Mitochondrial initiated apoptosis is a prevalent and well-studied area that contributes to muscle atrophy in aging. Inflammation is also linked to mitochondrial apoptosis pathways [8,10,46,202,203,204,205,206,207,208,209]. Normally, the release of mitochondrial contents to the cytosol indicates mitochondria that are not healthy. This release initiates a caspase cascade [8,46]. However, when caspases are reduced in mitochondrial mediated apoptosis, mtDNA triggers the innate immune Cyclic GMP-AMP synthase (cGAS), which is a DNA sensor that activates innate immune responses by producing a second messenger, cyclic GMP-AMP (cGAMP), and the stimulator of interferon genes (STING) [210,211,212,213,214]. The cGAS/STING pathway regulates dying cells to secrete type I interferon [215,216]. Inflammation resulting from either exhaustive exercise or mtDNA mutation is rescued by the concurrent loss of STING [217], while STING agonists increase STAT3-mediated immunosuppression [218,219]. A summary of several of the markers and genes associated with mitochondrially regulated inflammation are shown in Table 1. Together these findings suggest that the mitophagy signaling and the cGAS-STING pathway have important roles in regulating the inflammatory responses in muscle that can contribute to muscle loss and impaired muscle regeneration after an injury.

### 3.2. Metabolism in MSCs

#### 3.2.1. Mitochondria in MSC Proliferation and Differentiation

Myoblasts utilize glucose as a primary substrate, rather than mitochondrial regulated oxidative phosphorylation, and hyperglycemia increases MSC proliferation [220]. Glucose metabolism appears to be a driver for pyruvate dehydrogenase (PDH) mediated histone acetylation via acetyl-CoA production [221]. However, hyperglycemia also impairs mitochondria function while preventing cell progression from the S and G2/M phase which drives proliferation and disrupts normal differentiation and growth of muscle cells [220]. Nevertheless, this is a fine balance because glucose is able to be utilized as a substrate for oxidative phosphorylation in differentiating MSCs [221], and the restriction of glucose can inhibit MSC differentiation [222].

In addition to the proper balance of substrates to drive MSC metabolism during proliferation and differentiation, MSCs undergo decreased acetylation, in part via the regulation of the deacetylase SIRT1 [223], as part of the metabolic transition and mitochondrial utilization of glucose in differentiation. Pyruvate stimulates aspartate synthesis, and aspartate synthesis is an essential role of mitochondria in cell proliferation [224]. Reduced pyruvate dehydrogenase (PDH) activity and terminated histone acetylation must occur so that proliferating MSCs can exit the S phase and differentiate [221]. Thus, it has become clear that mitochondria and mitochondrially regulated metabolism have critically important roles in regulating MSC differentiation and growth in response to stimulation or injury. Furthermore, the disruption of mitochondrial respiratory function appears to block MSC differentiation [225]. These data emphasize the essential nature of having an adequate supply of healthy mitochondria for MSC differentiation during the program of muscle regeneration.

#### 3.2.2. Immunometabolism in Muscle Injury and Repair

Immunometabolism describes the close relationship between systemic and cellular metabolism and the immune system [226,227]. As mitochondria have an important role in regulating immune cell function [228], and mitochondrial dysfunctions regulate metabolic diseases [229] as well as contribute to muscle wasting conditions including sarcopenia, it is not surprising that mitochondria health and immune function are linked [230].

##### Exopher-Macrophage Regulation of Dysfunctional Mitochondria in Injury

The elevation of M1 pro-inflammatory macrophages in adipose tissue promotes metabolic dysfunction through increasing the cytokine levels of TNF-α, IL-1β and IL-6 [230,231]. Fgr activation in macrophages is associated with increased mitochondrial complex II activity and complex I degradation leading to pro-inflammatory macrophage polarization [230,232,233].

It has been proposed that enhancing mitochondrial metabolism by acute exercise improves macrophage function and reduces systemic inflammation through a decreased ROS-induced inflammatory response [234]. An additional link between dysfunctional mitochondrial metabolism and macrophages has been reported in cardiomyocytes. Cardiac macrophages surround cardiomyocytes and engulf dysfunctional mitochondria. The captured mitochondria are eliminated from the cytoplasm to the cellular milieu [235,236] in subcellular particles called exophers. Exophers are then eliminated by cardiac macrophages, which maintains cardiomyocyte homeostasis [235,237]. Notably, Mertk has been identified as the macrophage phagocytic receptor of exophers [236]. A knock down of Mertk or an age-associated reduction in Mertk impaired muscle recovery after a virus-induced physiological injury [238]. Thus, exopher regulation of dysfunctional mitochondria to M1 macrophages could play an important role in regulating muscle homeostasis and proteostasis during muscle injury and the initiation of muscle repair. The role of mitochondria in immunometabolism in muscle has been reviewed in detail [234].

##### Extracellular Vesicle Regulation of Mitochondria in Physiological Injury and Repair

Extracellular vesicles (EVs) could also play an important role in the regeneration of physiological or pathologically injured muscle cells by interfacing with both mitochondria and macrophages. EVs are lipid membrane encompassed particles shed from cells that were originally thought to be solely a means to eliminate cellular debris [10]. However, more recent observations have shown that EVs can transport proteins, mRNAs, microRNAs (miRNAs) and mitochondrial components in biofluids and blood to local and distant cells, which can maintain tissue homeostasis or induce pathology [239,240,241,242,243,244]. Although the metabolic functions of mitochondria are well known, the importance of mitochondria in mediating stem cell activity has not been widely appreciated because most stem cells have low mitochondria abundance and are largely dependent upon glycolysis, especially for proliferation [245]. Recent evidence suggests that crosstalk between mitochondria and other cell types occurs via circulating and local mediators. An intriguing new paradigm in cell-cell communication is that EVs (exosomes and microvesicles) may potentially provide a mechanism for sharing mitochondrial regulators (e.g., miRNAs, metabolites) or even whole mitochondria between cells.

**Figure 3:**
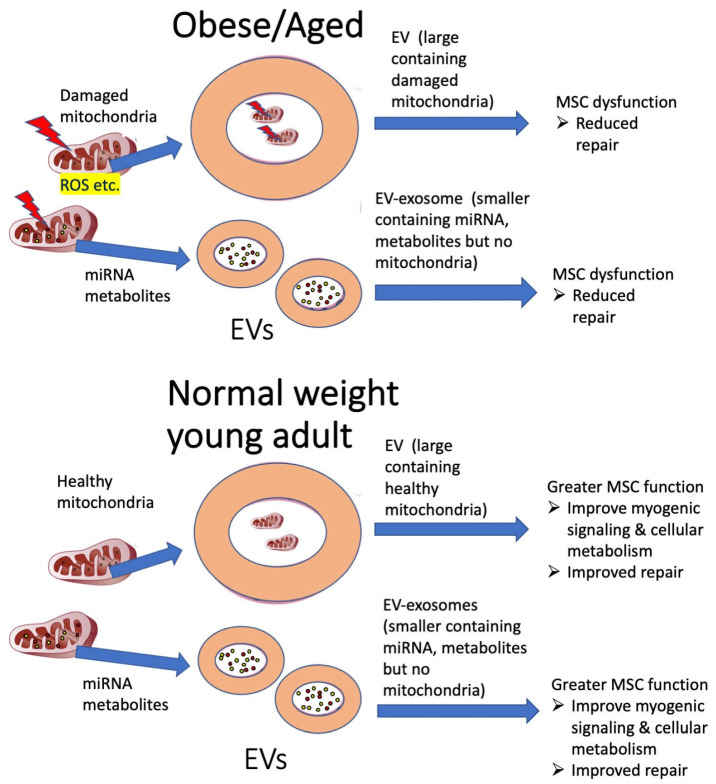
Hypothetical model for EV transport of damaged (obesity) and healthy (normal weight) mitochondria or mitochondrial metabolites, and other cargo to MSCs in obese muscle.

In support of this idea, EVs from highly proliferative cancer cells have been found to contain MT-CO2 and COX6c that are encoded by the mitochondria and nuclear genomes, respectively [246]. Furthermore, mitochondria have been identified in EVs from cardiomyocytes after an LPS challenge [247] and in EVs from mesenchymal stem cells after exposure to oxidative stress [248], although these are not likely healthy undamaged mitochondria. In addition, cancer cachexia [10], muscle damage [249], and obesity [250] have been reported to change the profile of EVs. Furthermore, each of these conditions has been shown to drive an increase in EV production. This is important because it is very likely that changes in the EV profile and abundance will impact intracellular communication and alter mitochondrial signaling. This raises the possibility that circulating EVs may have an important role in regulating mitochondria and in transporting mitochondria and its enzymes between cells. However, additional studies are needed to determine (a) if healthy mitochondria shuttling occurs in EVs or if this is solely for eliminating dysfunctional mitochondria (i.e., mitophagy); (b) if EV shuttling of healthy or dysfunctional mitochondria or its metabolites occurs in metabolic diseases such as obesity and/or after skeletal muscle damage; and (c) if the interaction of EVs that contain mitochondria or mitochondrial fragments participates in the regulation of macrophages in injury and muscle repair in a way that is similar or different from exophers. A hypothetical model for EV transport of damaged and healthy mitochondria in metabolic disease (obesity) and aging, and the potential impact on MSC function is shown in Figure 3.

### 3.3. Mitochondrially-Induced Oxidative Stress in Muscle Injury

#### 3.3.1. Mitochondria Associated Oxidative Stress as a Negative Regulator of Regeneration Following Muscle Injury

In addition to generating adenosine triphosphate (ATP) production through OXPHOS (Figure 2), mitochondria are also important for regulating programmed cell death via apoptosis [46], the production of reactive oxygen species (ROS) [251], and controlling muscle mass and muscle regeneration [54]. It is well known that mitochondria are damaged during different forms of skeletal muscle injury [88,252,253]. Damaged mitochondria have an elevated production of ROS [135,254,255], and produce less ATP for anabolic signaling, which interferes with muscle repair [256]. The elevation of ROS further magnifies oxidative stress, subsequently damaged mitochondria largely degenerate [253] leaving an energy vacuum, until the mitochondrial pool is repopulated. Traumatic muscle injury damages mitochondria [253], which can cause leakage of their contents into the cytoplasm, triggering cell death [46], elevating ROS [257], increasing cytoplasmic calcium accumulation, and causing endoplasmic reticulum (ER) stress. Furthermore, elevated ROS accumulation from damaged mitochondria [56,258] lowers mitochondrial “quality,” and induces a greater ratio of unhealthy to healthy mitochondria [259,260] that together, reduce the available energy. These changes can suppress anabolic signaling and delay the restoration of neuromuscular structure and function after an injury [256]. While antioxidants may facilitate tissue repair [257] and improve muscle function [203,261], this approach as a sole treatment for injury suppression of atrophy is only partially successful [3,262]. For full restoration there is a need to replace damaged mitochondria with healthy ones, which would correct the energy vacuum and, potentially, the mitochondrial modulated signaling that is present with incomplete mitochondrial regeneration. Injury markedly enhances mitophagy, which eliminates damaged mitochondria, thereby leaving a healthy pool of mitochondria to improve muscle repair [88]. It is likely that excessive ROS accumulation and dysfunctional mitochondria, along with poor mitochondrial turnover and reduced mitophagy, decrease MSC function with aging [263,264]. These outcomes negatively impact the ability of muscle to recover from injury or disuse [265]. This means that muscle repair after injury is impaired in aging in part because excessive mitochondrially regulated ROS negatively impact MSC function.

#### 3.3.2. Mitochondrial Reactive Oxygen/Nitrogen Species for Protecting Muscle against Injury and Improving Recovery

While earlier studies focused on the negative role of ROS on muscle, including muscle repair, more recent evidence has indicated a complex role for ROS regulation, including beneficial adaptations in muscle. ROS may regulate redox signaling pathways that control exercise-induced cellular responses as well as adaptations, including mitochondrial biogenesis, mitophagy, mitochondrial dynamics, antioxidant defense, and inflammation [266]. While ROS/RNS increases in the early stages of muscle injury and the onset of muscle repair, subsequent increases in antioxidant enzymes are required in repairing muscle to facilitate full recovery of muscle mass and function [135]. It is well established that during aging, a number of muscle fibers lose their functional innervation, leading to significant denervation and fiber degeneration and atrophy that further impair and, in some cases, prevent recovery after an injury. Although excessive ROS/RNS occurs as a response to mitochondrial damage in injury or denervation [13,267,268,269], a number of observations indicate that ROS and RNS play a role in pathways that regulate adaptations in muscle that protect against additional damage and/or improve recovery after an injury. For example, there is an age-related increase in ROS and RNS levels in muscle fibers during contractile activity in aging, and this was associated with an increase in muscle eNOS [55,262,270]. Muscle proteins from old mice also showed an increased 3-NT content. Inhibition of NOS indicated that NO decreased superoxide bioavailability in muscle mitochondria, although this effect was not age-related [271]. Thus, increased NO in muscles of old mice was associated with an increased 3-NT content that may potentially contribute to age-related degenerative changes in skeletal muscle [271].

Aged muscle also has a degree of denervation/reinnervation; denervation is associated with a large increase in ROS. Denervation models in rodents have been used to investigate the mechanisms leading to rapid declines in muscle mass and function following the loss of innervation [13,267,268,272,273]. Studies using this denervation model in rodents point to a role of mitochondrial dysfunction leading to oxidative stress in the mechanisms of denervation-induced muscle atrophy [13,268,274,275]. In addition, both voluntary wheel running and a cocktail of mitochondrial-targeted nutrients improve muscle regeneration after disuse [73,276], suggesting mitochondrial function and/or increasing mitochondria number can facilitate tissue repair.

The signaling role that ROS has in response to alterations in redox homeostasis includes the activation and inactivation of transcription factors, membrane channels and metabolic enzymes, in addition to regulating functional changes in calcium-dependent and phosphorylation signaling pathways [277,278].

##### Hydrogen Peroxide

Reactive oxygen species (ROS), and specifically hydrogen peroxide (H_2_O_2_), has been proposed to be a key factor in stimulating an adaptive change in contracting skeletal muscle [279,280,281,282,283]. H_2_O_2_ plays a key role in cell signaling and is usually formed within the mitochondria by the dismutation of superoxide generated from the electron transport chain. Some of these adaptations would be expected to provide some degree of protection against muscle injury and/or improve recovery from injury. Unfortunately, in the context of aging, many of these beneficial ROS-regulated adaptations to muscle are not evident, because elderly people are not as active and cannot exercise to the same intensity and duration as their younger counterparts. Previous studies have shown a significant increase in the mitochondrial generation of H_2_O_2_ and other peroxides in exercised or physiologically injured muscle fibers. This increase experimentally in rodents occurs 7 days after denervation and is sustained up to 21 days following muscle denervation. The mitochondrial electron transport chain is known to be a major source of cellular oxidative stress [284,285,286,287,288], and multiple studies have reported that ROS production was elevated from mitochondria leading to atrophy in denervated skeletal muscles including aged muscles [13,273,274,275,289]. Furthermore, NADPH oxidase 2 (Nox2) increases markedly in denervated muscle [290]. Elevated ROS levels were also associated with significant adaptations in the content of several proteins involved in the protection of cells against oxidative damage [290,291]. For example, the muscle content of heat shock protein 70 (Hsp70) and constitutively expressed Hsc70 were increased following denervation and remained elevated for up to 21 days post-denervation, the same time course as H_2_O_2_ elevations with denervation [290]. We have previously found that increases in Hsp70 occurred concurrently with exercise adaptations in aged muscles [123,292]. Together, these findings are consistent with the possibility that the increased Hsp70 and Hsc70 may play a role in maintaining protein homeostasis and preventing protein breakdown in response to ROS signaling in the denervated muscle [290,291]. Furthermore, prolonged muscle denervation also increases the abundance of Hsp25. The increased Hsp25 together with the changes in GPx1 are important in enhancing resistance to H_2_O_2_ damage in skeletal muscle fibers following denervation [293], and would also be expected to carry the same benefit to injury repair where the injury-induced nerve damage to the muscle. In addition, while the mitochondrial antioxidant MnSOD content in muscle did not change with denervation, TrxR2 was significantly increased at 3 and 7 days post denervation, and then declined, but the GPx1 content was significantly increased acutely and remained elevated throughout the 21 day study after denervation [290].

Interestingly, aging muscle appears to be partially protected against H_2_O_2_ damage in denervated muscle because mitochondrial peroxide generation was shown to be elevated in resting muscle from old (26 month) mice compared with adult (6–8 months) mice; but no age functional effect on muscle fiber H_2_O_2_ in vivo was seen [275,291]. Furthermore, although denervation increased mitochondrial release of H_2_O_2_ this did not appear to raise cytosolic H_2_O_2_ levels in aged muscles [291]. Thus, aging appears to have developed an adaptative response to high basal mitochondrial release levels of H_2_O_2_ as a protective event to slow atrophy and other muscle deterioration induced through denervation that also increases mitochondrial peroxide generation. In addition, denervation, which is a physiological type of injury, was associated with a significant increase in the muscle content of proteins involved in the potential generation of peroxides including Prx6 and cPLA2, which may be involved in the activation of NADPH oxidase. Together the data show that a mitochondrially-induced ROS increase was associated with an elevation in the content of Gpx1, TrxR2, and HSPs that are involved in the protection against oxidative damage and play important roles in the maintenance of redox homeostasis and proteostasis. Together, these data are consistent with the hypothesis that the increase in peroxide production following denervation may stimulate adaptations to protect the muscle fibers, which would be expected to facilitate muscle repair after an injury. However, a chronic and sustained increase in high levels of peroxide generation—such as that which occurs in muscle and other tissues at old ages—is still likely to overwhelm the adaptive responses of ROS in muscle, and activate catabolic processes that lead to degeneration, muscle atrophy, and poor recovery after a pathological or physiologically induced muscle injury such as overuse.

##### Molecular Hydrogen

While mitochondrial derived H_2_O_2_ can be destructive in high doses, molecular hydrogen (H_2_) has the potential to be anti-inflammatory and have antioxidant biological properties. H_2_ can react with hydroxyl radicals that are generated in the mitochondria, to protect cells from oxidative stress; however, the beneficial effects of H_2_ have not been fully studied because it is not generally thought to be metabolized in vivo.

Recently, adipose derived stem cells (ADSCs) have been examined as a potential source of stem cells which could be used to supplement MSCs in muscle repair; however, their viability is low in vivo. The utility in supplementing muscle repair with ADSCs would be better if their survival could be improved. Yang et al. [294] have shown that H_2_ provided to ADSCs significantly decreased mitochondrial ROS levels, increased the number of mitochondria, and promoted mitophagy, thus enhancing the survival and myogenic differentiation of ADSCs. This finding supports the idea that H_2_ provided to skeletal muscle myopathies or other pathologies might improve mitochondrial dysfunction and improve muscle repair after a pathological injury [294].

##### Mitogen-Activated Protein Kinases (MAPK)

Kinases such as p38-MAPK and JNK are involved in many stress responses, including insulin signaling and skeletal muscle contraction [295,296,297], and pathological injury [298,299,300]. Both p38-MAPK and JNK kinases are activated by high H_2_O_2_ in vitro. p38-MAPK is an important regulator of myogenesis and muscle repair [301]. Furthermore, the inhibition of p38-MAPK inhibits muscle stem cell differentiation [302,303,304,305,306] which would impair muscle recovery after a pathological injury. Thus, mitochondrial ROS induced p38-MAPK could be expected to facilitate improved repair after a pathological injury.

##### Peroxisome Proliferator-Activated Receptor Gamma (PPAR-γ)

PPAR-γ is a major regulator of mitochondrial genes and lipid metabolism, and it is a transcription factor for several genes controlling lipid storage/lipogenesis, energy expenditure, and the mitochondrial ATP generating OXPHOS pathway. PPAR-γ protein abundance and its transcriptional co-activator PGC1α are activated and increased in skeletal muscle by exercise [174,307,308]. Both PPAR-γ and PGC1α are modulated by increases in oxidative stress including NrF2 [309] and ROS [308,310]. However, an increase in PGC1α mRNA has been found in human muscle after supplementation with the mitochondrial targeted antioxidant MitoQ [311]. Thus, it is not clear if the ROS regulation of PPAR-γ and PGC1α is via direct oxidation or via redox-sensitive intermediaries [266,312].

##### Nuclear Factor-κB (NF-κB)

NF-κB is an important regulator of inflammatory responses. It is activated by contractile activity in skeletal muscle through the canonical NF-κB signaling pathway, which is mediated by IKK and IκB. NF-κB translocates to the nucleus where it acts as a transcription factor for genes involved in inflammation, stress, [313,314,315,316] and antioxidant proteins including catalase, thioredoxin (TRX), MnSOD and GPX [317,318,319,320,321]. H_2_O_2_ is known to regulate NF-κB signaling [322,323], p38-MAPK [324], and many other signaling molecules that regulate pathologies or regeneration [325,326,327,328,329]. However, it is important to note that the primary signaling molecules that have been examined are typically regulated in experimental conditions with relatively high H_2_O_2_ concentrations, typically in the range 10^−4^ to 10^−3^ M (i.e., 100 μM–1 mM)—much higher than levels experienced in vivo—so that the experimental relevance to physiological responses are less clear. Nevertheless, the role of ROS in regulating these genes is complex because H_2_O_2_ can activate NF-κB and MAPK signaling pathways can contribute to the inflammatory response, but they do not appear to induce cell injury, at least in intestinal porcine epithelial cells (IPEC-1) that are exposed to H_2_O_2_ [313,324]. Clearly, there is still much work to be done in understanding the roles of the physiological levels of ROS, including H_2_O_2_, in regulating molecules that modulate cell adaptations and muscle regeneration.

##### Nuclear Factor Erythroid 2-Related Factor 2 (Nrf2)

The nuclear factor erythroid 2-related factor 2 (Nrf2) and its target genes, such as heme oxygenase-1 (HO-1), provide protective mechanisms against high ROS levels that occur in disease, aging, or physiological or pathological injury. In addition, ROS levels are increased in muscle diseases such as Duchenne muscular dystrophy (DMD), leading to oxidative damage to the contractile proteins [330]. The disruption of Nrf2 signaling increases age-related vascular disease and tissue disruption [331]. However, exercise, which increases mitochondrial produced ROS, also increases Nrf2 expression. Nrf2 expression (ranging from 0.86 ± 0.4 to 1.76 ± 0.8) and GPx activity were reported to significantly increase after exercise intervention in humans [332]. These data suggest that exercise may induce Nrf2 activation, but whether this is a direct or indirect ROS regulation of Nrf2 by exercise requires additional work [332].

##### Sestrin2

**Figure 4:**
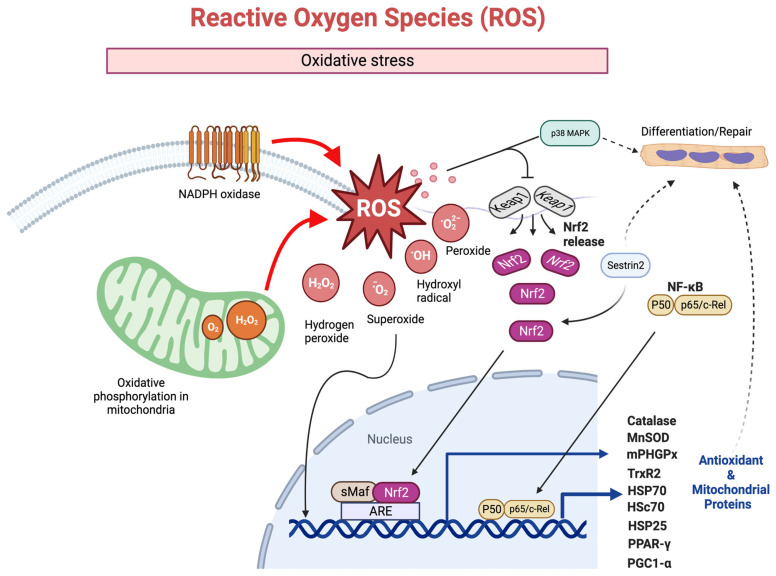
Overview of Reactive oxygen species (ROS) impacts on protective and myogenic signaling. Mitochondrial sources of ROS (e.g., H_2_O_2_) combine with non-mitochondria (e.g., NADPH oxidase) to contribute to cellular ROS. While excessive ROS is destructive, ROS signaling can also contribute to Keap1/Nrf2 mediated transcription for antioxidants. Likewise, NF-κB can induce stress and atrophic gene expression but also increase antioxidant genes (e.g., MnSOD, GPx, TRX). ROS (e.g., H_2_O_2_) can also directly activate transcription of antioxidant and mitochondrial genes. ROS induced activation of p38-MAPK can increase myogenesis while Sestrin2, working likely through Nrf2, can also contribute to myoblast differentiation. Myogenesis and differentiation are necessary elements of muscle repair after an injury. Thus, moderate ROS levels can contribute to improved regulation of muscle regeneration.

Sestrin2 is a stress-inducible protein that plays a critical role in the response to ischemic and oxidative stress. Normally, Nrf2 is inactivated, and it is bound to its repressor Kelch-like ECH-associated protein 1 (KEAP1) [333]. Sestrin2 can act as a positive regulator of Nrf2 by promoting the SQSTM1/p62-mediated autophagic degradation of KEAP1 [334,335]. Sestrin2 overexpression was found to suppress cell inflammation and oxidative stress, and to activate AMPK/Nrf2 signaling [336]. Sestrin2 signaling appears to work through Nrf2 [337]. Sestrin 2 also protects the heart against ischemic insults by reducing the generation of mtROS [338,339,340]. It is interesting that Sestrin2 expression follows a similar pattern to myogenin, and peaks approximately 48 h after differentiation, then decreases C2C12 mouse myoblast cells [341]. Whether Sestrin2 directly plays a role in myogenic differentiation is currently unknown. However, if this is the case, mitochondria oxidative stress, including the elevation of Sestrin2, could play a role in regulating the differentiation of myoblasts into muscle fibers during repair after a pathological injury. An overview of some of the ROS mediated signaling that contributes to cell protection and improved regeneration after a pathological or physiological injury is shown in Figure 4.

### 3.4. Alterations in Mitochondrial Genes during Injury and Repair

#### 3.4.1. Molecular Alterations in Mitochondrially Related Genes with Injury

Several changes in genes and proteins related to mitochondria function have been described after injury, although the mitochondrial expression changes that occur during repair after an injury have been less well studied (Table 2). Exercise-induced physiological injury has been shown to lower PGC1α mRNA and mitochondrial transcription factor A (TFAM), which are regulators of mitochondrial biogenesis. Solute carrier family 25-member 4 gene coding for adenine nucleotide translocase-type 1 (ANT1) was significantly higher in exercise-damaged muscle as compared to the control muscle [342]. The expression of muscle creatine kinase (CKm) mRNA was not significantly different between groups, whereas mitochondrial creatine kinase (CKmt2) was higher in damaged muscles. No significant difference was found for citrate synthase mRNA and, although not significant, there was a trend towards an increase of SOD2 mRNA, which is a marker of antioxidant capacity [342].

TBC domain family member 15 (TBC1D15) mRNA/protein levels were found to be downregulated in ischemic injured muscle [343]. It is known that TBC1D15 participates in the regulation of mitochondrial homeostasis, at least in part through maintaining the mitochondrial-lysosomal contacts [343], which presumably will improve mitophagy during muscle repair.

Connexin43 gap junction gene GJA1 has been shown to increase in response to ischemia. GJA1 is localized to mitochondria where it recruits actin to the mitochondria to support it, and induces mitochondrial fission independently from Drp1 and therefore protects mitochondria from further damage [345].

Mitochondrial associated ATF6 and GRP-78 were elevated in response to ischemic injury, which indicates that endoplasmic reticulum stress is subsequently associated with mitochondrial dysfunction [349]. Similar to ischemic injury, aging muscle, denervation, and loading, all increase cytosolic levels of mitochondrial DNA; cytochrome c is also elevated after injury, and tissue markers for apoptosis including Bax are increased in loading-induced injury [46,268,349,356,357].

#### 3.4.2. Molecular Alterations in Mitochondrially Related Genes in Repair/Myogenesis

PGC1 and estrogen-related receptor (ERR)-induced regulator muscle 1 (PERM1) is relatively high in skeletal and cardiac muscle mitochondria and is transcriptionally regulated by PGC1α and ERR [358]. Perm1 increases mitochondrial biogenesis and reduces cell death [346,347,348].

Nicotinamide adenine dinucleotide (NAD^+^) plays a central role in muscle metabolism and is an important co-factor for the tricarboxylic acid (TCA) cycle and OXPHOS. Thus, NAD^+^ is important for providing cellular energy metabolism for muscle regeneration after a pathological injury [344]. NAD^+^ levels decline with aging and muscle pathologies including muscle pathological or physiological injury, which contributes to reduced energy availability for muscle repair. NAD^+^ synthesis from nicotinamide riboside (NR) requires nicotinamide riboside kinases (NRKs) 1 and 2 to phosphorylate NR to NMN. NMN is increased during primary mouse myoblast differentiation in vitro, with nicotinamide riboside kinase 2 (Nmrk2) mRNA expression peaking during the time of myoblast differentiation [344]. Nmrk2 is a damage-inducible transcript in [344] muscle and even in non-muscle tissues (i.e., during neuronal physiological injury) [359,360,361,362,363].

CTX-induced pathological injury to the gastrocnemius muscle in old mice, followed by muscle repair, was found to have greater mitochondrial Complex III activity and reduced ATP synthase activity [59,350] in the muscle. In addition, mitochondrial Complex I, III and ATPase activity, along with SOD1 and catalyase abundance, have been reported to increase in repairing muscle after CTX injury [350]. In addition, mitochondrial fission 1 (Fis1) mRNA has been shown to increase over 5 days of recovery after a freeze-induced pathological injury to skeletal muscle [351,352]. Fis1 and mitochondrial biogenesis-related genes including PGC1β, PRC, NRF-1, NRF-2, and TFAM increased throughout muscle regeneration after freeze-induced pathological injury. Mitochondrial single-stranded DNA binding protein 1 (mtSSB), a regulator of replication, repair, and recombination of mitochondria, was also elevated in regenerating skeletal muscle tissue, and remained above the levels found in undamaged/control tissue for up to 28 days of repair after CTX injury. The expression of genes involved in mitochondrial fission was increased during the early phases of muscle regeneration, whereas the expression of the genes involved in mitochondrial fusion increased later during muscle regeneration [351,352]. Furthermore, it is important to note that muscle regeneration was delayed while pharmacologically blocking mitochondrial protein [351] synthesis. This indicates that mitochondrial biogenesis and health are key elements in successful muscle regeneration after a pathological injury.

Consistent with these results, the mitochondrial fission protein dynamin 1-like (DRP1) is elevated 14 days following CTX-induced skeletal muscle damage [353,354]. Furthermore, activated ULK1, BCL1 interacting protein (BNIP3), and MAP1LC3-II were elevated 14 days post-CTX induced injury in regenerating muscle, even when the animals were treated with the autophagy inhibitor 3-methyladenine (3-MA) [353,354]. Additional work showed that there was an increase in mitophagy proteins, including Drp1, BNIP3, Pink1, and Parkin that accompanied mitochondrial localization of MAP1LC3B-II in repairing muscle 7 days after a freeze pathological injury [88,355].

### 3.5. Mitochondrial Dysfunction and Cellular Regeneration in Myopathies

Muscle pathological injury and signaling for myopathic muscle deterioration often share common pathways. For example, Duchenne muscular dystrophy (DMD) is a progressive muscle wasting disease resulting from loss of the dystrophin gene that results in muscle sarcolemma stiffness and susceptibility to damage and muscle degeneration [364]. DMD is characterized as an insufficient regeneration after muscle damage [364]. However, like muscle pathological injury, mitochondrial dysfunction can also play an important role in the progression of muscle degeneration in myopathies including DMD.

While many studies have addressed the problem of partially restoring or attempting to fully restore the dystrophin gene [365,366,367], most of the strategies have been only partially successful. However, the targeting of secondary pathological mechanisms or muscle repair after contractile catabolism, provides an important strategy to improve function while gene replacement therapies are being optimized. Several studies have pointed to mitochondrial dysfunction, including reduced ATP production, reduced mitochondrial biogenesis, ion-induced dysfunction, and ROS as having important roles in regulating mitochondrial dysfunction that contributes to muscle degeneration in DMD.

#### 3.5.1. Oxidative Enzyme Loss and Mitochondrial Dysfunction in DMD Muscle Degeneration

Defects in mitochondrial enzymes of the tricarboxylic acid cycle in DMD respiratory chain complexes [368] account for a lower maximal rate of respiration [369]. Mitochondrial dysfunctions are one of the earliest deficits that have been reported in DMD and, as such, 50% of the ATP content [370,371] is present in DMD muscles as compared to non-myopathic aged matched muscles, which is at least in part, a result of reduced Complex I activity [372].

#### 3.5.2. Mitochondrial Molecular Dysfunction in DMD Muscle Degeneration

Mitochondrial dysfunction and the loss of ATP production have a direct impact on muscle fiber degeneration [373,374,375]. The accompanying progressive mitochondrial biogenesis impairment is associated with increased deacetylation of the promoter for PGC1α. Histone deacetylation is inhibited by givinostat, that positively modifies the epigenetic profile of the PGC1α promoter. This sustains mitochondrial biogenesis and a fiber type switch towards oxidative fibers. Increases in the activation of SIRT1 and PGC1α through resveratrol have been shown to improve mitochondrial function in DMD [376,377]. Givinostat exerts relevant effects at the mitochondrial level, acting as a metabolic remodeling agent that is capable of efficiently promoting mitochondrial biogenesis in dystrophic muscle [373].

#### 3.5.3. Disruption of Ion Homeostasis and Mitochondrial Dysfunction in DMD Muscle Degeneration

Excitation-contraction coupling (ECC) deregulation and defective mitochondrial respiration are early responses to DMD which are followed by disrupted Ca^2+^ homeostasis, disruption of calcium buffering, and overloading mitochondria with excessive Ca^2+^ levels, which contributes to overall mitochondrial dysfunction [378,379,380]. The normalization of mitochondrial calcium and potassium homeostasis in the muscle by increasing the calcium retention capacity and reducing mtPTP opening reduces ROS and improves mitochondrial ultrastructure and function [381,382,383].

Previous studies have also suggested that there is a link between changes in the intracellular ROS levels that lead to increased cytosolic Ca^2+^ and the appearance of Ca^2+^ sparks in mammalian skeletal muscle [380]. These greater Ca^2+^ signals contribute to mitochondrial Ca^2+^ accumulation in mouse dystrophic (mdx) muscle fibers and accelerated mitochondrial ROS production [380]. These results suggest that the excessive ROS production and the simultaneous activation of abnormal Ca^2+^ signals intensify each other, which together amplifies the muscle pathological injury cycles in DMD muscle [380].

A reduction in the expression of sarcolipin (SLN), which is an inhibitor of sarcoplasmic reticulum (SR) Ca^2+^-ATPase (SERCA), reduces the degenerative effects of muscular dystrophy in mice. This is because SERCA is the Ca^2+^ pump which removes cytosolic calcium and sequesters it in the SR. Lowering cytosolic Ca^2+^ reduces calcium-activated proteases and therefore attenuates muscle wasting in DMD [384].

#### 3.5.4. ROS and Mitochondrial Dysfunction in DMD Muscle Degeneration

Several different mitochondrial dysfunctions have been reported in the muscles of the dystrophin-deficient mdx mice [380,385,386]. Elevated ROS accumulation has been reported to occur in DMD in part as a result of elevated NADPH oxidase 4 (NOX4) in muscle stem cells [387]. Elevated iron levels have also been reported in dystrophic mouse muscle and this is accompanied by an elevated muscle ROS level [388]. Furthermore, higher ROS levels in DMD can increase the sensitivity of the mitochondrial permeability transition pore (mtPTP). The mtPTP is responsive to various stimuli, including ROS and calcium loading [389]. Premature or prolonged opening of the mPTP releases the mitochondrial contents to the cytoplasm, and this can amplify ROS and induce apoptosis [46,390,391]. Chemical stabilization of the mtPTP with TR001 has been shown to improve respiration of myoblasts and myotubes from DMD patients, suggesting that mtPTP-dependent dysfunction also occurs in the human disease [392].

#### 3.5.5. Dysregulation of K^+^ Homeostasis

Dysregulation of K^+^ homeostasis contributes to mitochondrial dysfunction in DMD. Mitochondrial dysfunction in the skeletal muscles of dystrophin-deficient mdx mice is accompanied by a reduction in K^+^ transport [379]. Furthermore, hyperactivity of the calcium-activated potassium channel type 3.1 (K(Ca)3.1) impacts macrophage phenotype and fibroblast proliferation, both of which are major contributors to inducing muscle damage and regulating muscle repair. In conclusion, this work supports the idea that K(Ca)3.1 channels play a contributing role in controlling damage-causing cells in DMD [393]. Dysregulation of K^+^ homeostasis is associated with a decrease in the expression of the mitochondrial large-conductance calcium-activated potassium channel in the muscles of mdx mice. Pharmaceutical normalization of the K^+^ flux reduces ROS production and improves muscle repair [379].

### 3.6. Mitochondria in MSC Proliferation

The proliferation of MSCs provides an initial step in expanding the myogenic stem cell line, and the suppression of MSC proliferation limits the myogenic cell pool for muscle regeneration [28,394,395]. Increased MSC proliferation is associated with increases in Paired box 7 (Pax7) acetylation [394], and Pax7 increases in abundance along with nuclear translocation of Inhibitor of differentiation-2 [396,397] and elevated c-Myc [398]. Other proteins may modulate MSC proliferation through CpG methylation [399], whereas reductions in c-Myc may limit muscle adaptations and regeneration [357,400].

**Figure 5:**
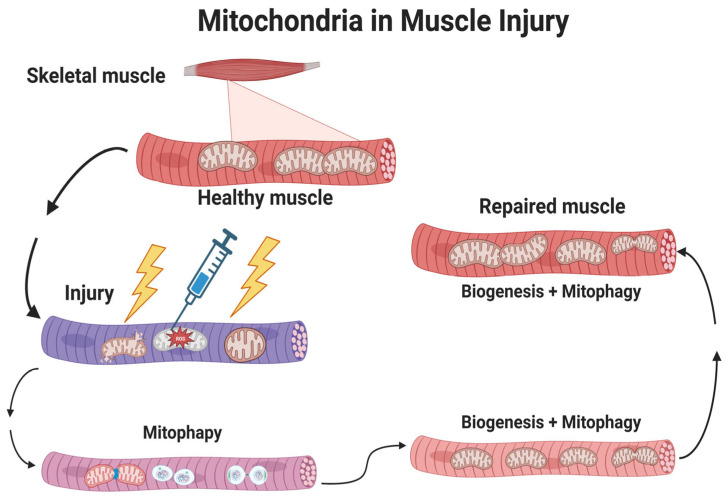
Mitochondrial cycles in muscle regeneration. Progress of MSCs proliferation and differentiation in response to pathological injury. Skeletal muscle can become injured from activity, or trauma or experimentally, undergo an acute inflammatory response driven by pro-inflammatory cytokines (e.g., TNFα, IL-6, IL-7) that coincides with an increase in mitochondrial fission and mitophagy. A conversion from the M1 to the M2 macrophage phenotype which participates in regeneration of muscle is regulated in part through withdrawal of the proinflammatory cytokines and increases in anti-inflammatory cytokines (e.g., IL-4, IL-10). Mitochondria biogenesis is initiated with increasing M2 macrophage and anabolic signaling, resulting in increased fusion contacts that increase mitochondrial size and number. The new mitochondria support the energy required for MSC differentiation, new protein assembly and repair of injured muscle. Thereafter, the fiber hypertrophies while there is a continuation of mitochondrial remodeling that includes a balance between mitochondrial biogenesis and mitophagy.

Increasing mitochondria biogenesis appears to be an important strategy to protect myoblasts against damage in response to excessive ROS and cell death [401]. Furthermore, mitochondrial biogenesis is necessary for optimal muscle regeneration (Figure 5), and enhancing mitochondria biogenesis improves overall muscle repair [402]. Resveratrol is an activator of Sirtuin 1 (SIRT1), a NAD^+^ sensitive deacetylase. Supplementation with resveratrol protects mitochondria against ROS-induced damage and apoptosis [202], and increases PGC1α, leading to elevated mitochondria biogenesis [41,402]. The activation of SIRT1 via resveratrol also improves muscle repair after loading [41] or pathological injury [3,41,59,402]. While not a universal finding [403], SIRT1 and resveratrol as an enhancer of SIRT1-mediated mitochondrial biogenesis, do not appear to improve MSC proliferation; rather, a loss of SIRT1 has been shown to improve MSC proliferation in aged muscles [59]. This may be due in part to suppressing the deacetylase activity of SIRT1, and thereby permitting a greater level of acetylated myogenin [394]. Furthermore, quiescent MSCs have a low number of mitochondria, which are small-sized (i.e., 90% are <0.5 μm^3^), with only 5% of mitochondria slightly larger-sized (0.5–4 μm^3^ [171]). However, one day after a CTX pathological injury, during the point of high inflammation, MSCs were reported to have more abundant, larger, and rounder mitochondria, which suggested increased mitochondria fusion and potentially an acute increase in mitochondria biogenesis [171]. Interestingly, 3 days after pathological injury, during the period of heavy MSC proliferation, MSCs contained a higher number of small, more spherical mitochondria as compared to one day post pathological injury, which suggests that mitochondrial fission occurred during MSC proliferation [171]. Recent data suggest that increasing mitochondrial fission, where mitochondria are divided into smaller components and then are targeted for autophagic removal (mitophagy), may increase MSC proliferation [71,404]. To test this idea, Hong et al. [171] used a knockout of the mitochondrial fission protein dynamin-related protein (DRP1), and showed that MSC expansion was reduced when DRP1 was lost, and elevated mitophagy to remove mitochondria could promote MSC proliferation. This suggests that while mitochondrial biogenesis may have an initial role during the inflammatory phase of muscle repair, increasing mitochondria abundance is not an important component that is required for the expansion of the MSC pool when MSCs are proliferating (Figure 5). However, mitochondria abundance may instead be very important to support MSC differentiation and remodeling of muscle in later periods of muscle regeneration after a pathological injury. Thus, mitochondrial dynamics play a changing but important role in the expansion and differentiation of MSCs in response to a pathological injury [404].

### 3.7. Mitochondria in MSC Differentiation

The downregulation of Pax7 and an elevation of myogenin promotes differentiation of myoblasts into myotubes [405,406]. It also promotes myogenin stability and an oxidative muscle phenotype [407,408]. mTOR is also important for regulating the protein synthesis required for differentiation [409]. Several modulators of myoblast differentiation have been identified, including a role for M-cadherin-mediated signaling, which attenuates β-catenin phosphorylation at Ser31/37/Thr41 by GSK-3β; this regulation through myogenin has a positive effect on myogenic differentiation induced by canonical Wnt signaling [410,411].

Another interesting differentiation regulator of myoblast to myotubes in muscle regeneration are mitochondrial adenine nucleotide transporters (ANTs). ANTs exchange ADP/ATP across the inner mitochondrial membrane [412] and make up a substantial proportion of the mitochondrial inner membrane protein [413]. The lack of the ANT2 isoform has a negative impact on mitochondrial function and cellular energy homeostasis, affects key signaling pathways that are essential for cellular remodeling and cell survival, and reduces differentiation of MSCs. Thus, ANT2 has a role in modulating the mitochondrial regulation of oxidative metabolism and MSC differentiation [414]. Differentiation of MSCs has a high energy cost, which means that there is a demand for mitochondria biogenesis to support protein assembly during differentiation and muscle repair (Figure 5).

#### 3.7.1. Mitochondrial Biogenesis in MSC Differentiation and Regeneration

As satellite cells/MSCs and myoblasts move from proliferation to differentiation, they also undergo a shift in metabolic substrates, which requires the modulation of mitochondria and mitochondrial function. The number and volume of mitochondria that reside within MSCs is also determined by the balance between mitochondrial biogenesis that generates new mitochondria, and mitophagy that eliminates mitochondria [45]. The expansion of the mitochondria pool that is required in differentiation results from an increase in PGC1α-mediated regulation and an increase in fusion of mitochondria resulting in larger mitochondria [171]. Thus, enhancing mitochondrial biogenesis increases MSC-regulated muscle regeneration [402], largely as a result of the impact of mitochondrial abundance in regulating differentiated MSCs and protein assembly.

Tumor necrosis factor (TNFα) and interleukin-6 (IL-6) are elevated in inflammation and polarize macrophages towards the M1 state [415,416]. Interleukin (IL)-7 is also known to enhance the M1 activity and infiltration of myocardial ischaemia/reperfusion (I/R) injury [417]. Advanced glycation end product (AGE)-induced activation through RAGE/TLR4/FOXC2 signaling participates in M1 polarization [418].

In contrast, the suppression of TNFα and IL-6 moves macrophages towards the non-inflammatory M2 state for muscle repair [415,416]. Interestingly, M2 macrophage-expressing IL-4 and IL-6 cytokines induce endothelial cell proliferation while IL-4 promoted proliferation of myoblasts and prevented myofibroblast-induced collagen type I secretion for improving muscle specific repair [419]. Furthermore, IL-10 appears to promote the M2 macrophage phenotype in muscle pathological injury so that the percentage of M2-like macrophages was increased dramatically while the M1/M2 macrophage ratio was reduced [416,420,421].

Cardiotoxin (CTX)-induced muscle pathological injury activates cytokines such as IL-6, IL-4, TNF-α, IL-33 and IL-10, with presumed roles in either activating M1 or M2 macrophages in regeneration. Transforming macrophages from the proinflammatory M1 into the M2 sub-type was also reported to be associated with TGF-β1/Smad3/p38/ERK1/2 signaling in muscle [422]. The suppression of M1 cytokine-induced inflammation also improves mitochondrial metabolism and increases PGC1α as an inducer of mitochondrial biogenesis while also decreasing ROS accumulation [423].

After CTX injury, old mice have a lower muscle inflammatory response compared to young mice, with a greater M2 macrophage recruitment and IL-10 expression [424]. The initial lower M1 phase reduced MSC proliferation after pathological injury, and reduced muscle regeneration. The temporal immune and cytokine responses of old mice were partially restored to a young phenotype following reconstitution with young cells (Y-O chimeras) [424]. Improved immune responses in Y-O chimeras were associated with greater MSC proliferation compared with O-O chimeras. These data show that a proper balance between the cytokine driven M1 and M2 macrophage response is needed to allow for optimal proliferation of muscle stem cells followed by differentiation of those cells for recovery of muscle mass and function after a pathological injury.

The M1 inflammation-driven MSC proliferation also coincides with enhanced mitochondrial fission and mitophagy, and this progresses to a state of predominant mitochondrial fusion, and biogenesis during differentiation, which is impacted by the requirement for greater energy production to facilitate MSC differentiation. Clearly, if MSC proliferation is blunted due to inadequate mitophagy, then the available pool of MSCs for myofiber differentiation and subsequently muscle regeneration will be compromised. However, if MSC proliferation is adequate, the proper differentiation and migration of MSCs are still required to complete muscle regeneration, and this requires an increase in mitochondrial size and number (Figure 4). Thus, mitochondria help to regulate this delicate balance between MSC proliferation and differentiation.

#### 3.7.2. Mitochondrial Fusion in MSC Differentiation

The combination of mitochondrial biogenesis and mitochondrial fusion promotes a greater volume of larger mitochondria, which elevates the total energy generation capacity in regenerated skeletal muscle [71]. In contrast, the inhibition of mitochondrial biogenesis and mitochondrial protein synthesis inhibits muscle regeneration in pathological injury models [71].

Optic atrophy-1 (OPA1) is an essential GTPase that is responsible for the fusion of the mitochondrial inner membrane. OPA1 connects the mitochondrial structure with the metabolic function of mitochondria. This link occurs when the membrane potential across the inner mitochondria membrane in MSCs (ΔΨ_m_) is normal, so that the long L-OPA1 isoform functions to induce fusion of the inner mitochondrial membrane [425]. Fusion of the membranes of two small mitochondria result in a larger mitochondrion. In contrast, when ΔΨ_m_ is lost, L-OPA1 is cleaved to a short, fusion-inactive S-OPA1 isoform by OMA1 which causes mitochondrial fragmentation [425,426]. Mitochondria in quiescent MSCs become fragmented by S-OPA1 upon an activation stimulus, which helps to drive the exit from cell quiescence to proliferation [427].

Assembling more and larger mitochondria becomes a dominate program in differentiation and cell growth after pathological injury [171,428]. Mitofusin 2 (Mfn2) is a mitochondrial protein that is important in mitochondrial fusion [428] for increasing mitochondria size. Mfn2 is also increased in response to pathological injury. Mfn2 remodeling to generate larger mitochondria is required for maturation and remodeling of injured myofibers [428], whereas regenerated fibers that contain central nuclei have increased levels of ROS and express neonatal myosin in the absence of Mfn2 [428]. An additional role of Mfn2 is to block Hypoxia-induced factor 1 alpha (HIFα), which is induced during muscle pathological injury [429]. Sustained HIFα expression blocks the transition of neonatal fiber growth to an adult phenotype, whereas blocking HIFα accelerates remodeling and muscle repair after pathological injury.

#### 3.7.3. Mitochondrial Mitophagy in MSC Differentiation

While mitochondrial biogenesis is critical for optimizing muscle regeneration, the degradation of damaged mitochondria that was not cleared during the period of MSC proliferation through mitophagy must be removed via mitophagy during differentiation to provide a strong healthy pool of mitochondria and meet the high metabolic energy demands [71]. Thus, while mitophagy is important in MSC proliferation, a balanced mitophagy program is still important in MSC differentiation [168]. Furthermore, Pink1 and Parkin, E3 ubiquitin-protein ligases that target mitochondria for mitophagy, are activated at the early phases of MSC differentiation [430]. In addition, the loss of Parkin impairs recovery from muscle pathological injury [431]. Moreover, the downregulation of Mfn2 results in increased dynamin-related protein 1 (Drp1) that together blocks mitochondrial remodeling and myoblast differentiation [430]. Presumably, an increase in mitophagy is needed to ensure the availability of a healthy pool of respiring mitochondria that will provide adequate ATP for mitochondrial biogenesis and protein assembly during differentiation and muscle repair after a pathological injury (Figure 4). Healthy mitochondria for this review are defined as mitochondria that are respiring normally (not “hyperactive” metabolically), do not have an excessive Ca^2+^ load, have normal mitochondrial dynamics (fission/fusion), have normal metabolism, and do not have an excessively open mitochondria permeability pore to allow the contents of the mitochondria to leak into the cytoplasm. 

#### 3.7.4. mRNA and microRNA Regulation of Differentiation Activates Mitochondrial Biogenesis

The MSC/satellite cell differentiation process is regulated by myogenic regulatory factors (MRFs), including MyoD, myogenic factor 5 (Myf5), MRF4, and myogenin. Myogenin is particularly important for regulating muscle differentiation [29] and it has been linked to the regulating of oxidative metabolism [407]. Several microRNAs (miR) are significantly upregulated during the differentiation of myoblasts and MRFs, and several have been implicated in regulating muscle fiber regeneration after pathological injury via mitochondria biogenesis [432]. Of note, the expression of miR-133a is increased during the differentiation of C2C12 myoblasts, and also promotes mitochondria biogenesis during differentiation. While a miR-133a mimic is sufficient to induce the biogenesis of mitochondria and differentiation of C2C12 myoblasts, a miR-133a inhibitor abolishes cell differentiation [432]. This suggests that the regulation of MSC differentiation during repair has several layers of control for muscle repair, yet all appear to involve mitochondria as a central piece in their pathways of control. There are other miRs that may have a role in mitochondria abundance, and likely many other miRs that have a role in regulating mitochondria biogenesis in MSCs that are yet to be discovered.

#### 3.7.5. Strategies for Attenuation of Mitochondrial Aging

Given the importance of mitochondrial abundance and health to the regenerative processes in muscle and proteostasis, one strategy for improving muscle regeneration is to enhance mitochondrial health in aging. Pharmacological strategies such as metformin have shown significant promise in improving mitochondrial health through mitophagy, and improving muscle stem cell function (reviewed in [433]). Other strategies to improve mitochondrial health are through nutrition and nutraceuticals, which have been used with varying degrees of success to attenuate mitochondrial aging. However, few of these strategies have been applied to muscle regeneration in aged model systems or humans. A comprehensive discussion of all of the pharmacological and nutritional strategies that have been applied to improve aging mitochondria is beyond the scope of this review, but a few nutritional/nutraceutical approaches have been identified below.

##### Resveratrol

Several nutraceuticals have been shown to improve satellite cell activation in culture and/or activation and differentiation in muscles of aged rodents. Resveratrol is a SIRT1 activator that also drives mitochondrial biogenesis through the mitochondria master gene PGC1α. High levels of ROS (e.g., H_2_O_2_) damage mitochondria and induce mitochondrial-regulated apoptosis signaling [267], and suppress SIRT1 protein abundance [202] and PGC1α. However, resveratrol not only reduced the loss of SIRT1 and reduced ROS-induced apoptosis by suppressing Bax, caspase-9, -8 and -3 activity in myoblasts, but it promoted myoblast survival [202]. Furthermore, resveratrol fed to aged rats [2,3] and aged mice [55] was found to reduce the abundance of muscle pro-apoptotic Bax and cleaved caspase 3, which improved muscle recovery after disuse followed by loading-induced physiological injury [3]. Resveratrol treatment has a protective effect against aging-induced oxidative stress in skeletal muscle, likely through the upregulation of MnSOD activity [262]. Furthermore, resveratrol added to exercise-training in elderly men and women significantly improved mean mitochondrial density, fiber area, and total myonuclei by 15.3%, 45.3% and 20%, respectively, in muscle fibers from the vastus lateralis of older subjects [41]. This suggests that improving mitochondria in older persons by resveratrol, through the SIRT1-PGC1α axis, was also associated with better muscle adaptations in skeletal muscle.

##### Beta-Hydroxy-Beta-Methylbutyrate (HMB)

HMB is a naturally occurring leucine metabolite that has the capacity to attenuate plantar flexor muscle loss and increase myogenic stem cell activation in repairing and adapting the muscles of old rodents [434]. Furthermore, HMB reduces mitochondria-associated apoptotic signaling, including a 40% reduction in Bax and a 12% reduction in cleaved caspase-3 in fast contracting plantaris muscle, and a 14% reduction in Bax and a significant 9% reduction in cleaved caspase 3 in slow contracting soleus muscles, as compared with vehicle-treated animals [205]. The suppressed mitochondrially regulated apoptotic signaling suggests that mitochondria are healthier in aged animals after HMB treatment, and this should provide a better platform for repair after a physiological injury. However, the benefits for HMB appeared to be limited to the loaded and/or injured muscles of old animals because HMB treatment did not alter control muscles in old rats that had not been stressed [435].

##### Epigallocatechin-3-Gallate (EGCg)

EGCg, an abundant catechin in green tea, has been shown to improve mitochondrial health by reducing apoptotic signaling and improving muscle recovery in response to reloading-induced physiological injury following disuse through hindlimb suspension. EGCg fed to old rats increased the expression of mitophagy/autophagy genes (e.g., ATG16L2, SNCA, TM9SF1, Pink1, PIM-2). Relative to vehicle treatment, EGCg treatment increased ATG12 protein abundance (36%) [436]. EGCg appeared to “prime” autophagy signaling and enhance autophagy gene expression and protein levels during unloading (atrophy and muscle damage) in muscles of aged rats, perhaps to improve the clearance of damaged organelles. EGCg treatment increased MSC proliferation and differentiation in plantaris and soleus muscles during recovery from hindlimb suspension, induced atrophy and damage compared with vehicle-treated muscles, and decreased oxidative stress, abundance Bax (−22%), and FADD (−77%) in the plantaris muscle of old rats [9,36,390]. However, EGCg suppressed autophagy signaling after reloading, potentially to increase the recovery of hindlimb muscle mass and function after loading is restored. Thus, while the responses are complex, the mitophagy/autophagy signaling during the period of damage, and the removal of the autophagic signals during muscle repair, suggest that the remaining mitochondria pool was healthier and restored function more fully than non-treated animals [436,437].

##### Sulforaphane (SFN)

SFN is a natural compound that has Nrf2-related activator functions and increases the expression of cytoprotective genes. Aging muscle has a significant drop in Nrf2 activity and mitochondrial functions along with decreased mitophagy [438]. SFN has been shown to restore Nrf2 activity, mitochondrial function, and the activation/differentiation of skeletal muscle MSCs to that of younger muscle [438]. This observation further supports the idea that mitochondria health underlies muscle health and the ability for satellite cells/MSCs to regenerate tissue after a physiological injury.

##### Nicotinamide Riboside (NR)

NR is a NAD^+^ precursor which is a partner for SIRT1 activation of PGC1α. Aging reduces NR, and this contributes to lower mitochondrial abundance. NR-treated mice exhibited enlarged slow-twitch fibers and a trend toward more slow fibers, more mitochondria and greater mitochondrial activity in both mouse and human myoblasts and human myotubes [439]. Additionally, NR treatment improved the differentiating capacity of myoblasts and increased myotube size and fusion index upon stimulation of these progenitors to form multinucleated myotubes. This observation provides additional support for a direct link between mitochondrial activity and health and myogenesis.

### 3.8. Mitochondrial-Nuclear Axis in Physiological Muscle Injury and Repair

Physiological muscle injury can occur as a result of many different conditions including, but not limited to, overuse, eccentric contractions, or downhill running (also eccentric damage). Typically, pathological muscle injury is thought to require MSCs to modulate muscle repair. However, surprisingly, muscle nuclei appear capable of migration to the sites of eccentrically damaged muscle to engage in repair even without recruiting and proliferating MSCs for the repair process [440]. The myonuclear migration is a chemotactic response to increased cytosolic calcium at the site of injury. Myonuclei-induced repair required increased transcription of contractile proteins. Furthermore, myonuclear movement to the injury site was linked to dynenin and microtubules, and this appears to be important for the local delivery of the transcripts for contractile protein assembly; but myonuclear migration was not a requirement for increased transcription and providing a contribution to muscle repair [440].

Another important observation in the repair process after physiological injury is that mitochondria also migrate to the site of injury and their job appears to be to buffer the excessive Ca^2+^ load in damaged muscle cells. If Ca^2+^ chelators were added to the damaged muscle, nuclear migration and repair was attenuated. However, if mitochondrial Ca^2+^ uptake was reduced pharmacologically with the calcium blocker Ru360, cytosolic Ca^2+^ remained high and nuclear migration to the injury site was suppressed, as was muscle repair [440]. Furthermore, reducing mitochondria Ca^2+^ uptake through a mutation in the mitochondrial calcium uniporter reduces contractile function and impairs muscle sarcolemma repair [441]. Thus, there is a link between mitochondrial abundance at the site of injury and muscle repair and Ca^2+^ uptake by those mitochondria. More work is needed to fully understand if the only role for mitochondrial migration and participation in injury repair is to buffer excessive Ca^2+^ and perhaps other ions at the injury site so nuclear transcription can occur appropriately, or if mitochondria contribute to repair in additional ways (e.g., generate ATP, anabolic signaling etc.). Nevertheless, this cooperation between myonuclei and mitochondria in muscle repair suggests the possibility of crosstalk between these two organelles. This would not be unexpected given that transcription of nuclear genes occurs in both the mitochondria genome and the nuclear genome [353], and mitochondrial biogenesis is dependent upon the import of precursor proteins originally encoded by the nuclear genome [442].

### 3.9. Mitochondrial Transplantation Increases Mitochondria Abundance and Improves Muscle Regeneration

Improving the number and activity of healthy mitochondria in old muscle appears to be important for maximizing muscle regeneration in aging. Graded exercise has been used for many decades as a primary rehabilitative approach to improve muscle repair and regeneration after a physiological injury in young or older persons. Exercise provides a stimulus to increase MSC/satellite cell proliferation [73], increase muscle capillarity [443,444,445] (to increase substrate availability for mitochondrial metabolism), increase mitophagy [45,49,446,447] (to improve the overall quality of the mitochondrial pool), decrease apoptosis [204], and increase mitochondria biogenesis in muscle [45,46,49,447,448,449]. Together, these mitochondrial adaptations lead to the improved recovery of injured muscle. However, loss of mitochondrial function with disuse, aging, and disease compromises the muscle’s capacity to regenerate after a physiological injury. Furthermore, exercise is not always possible for persons with severe injuries, prolonged bed rest which induces muscle disuse atrophy, the elderly, and diseased persons; so alternative approaches for improving healthy mitochondria in injured muscle should be considered. Given the importance of healthy mitochondria to regulate muscle repair, our laboratory has recently examined the potential that supplementing injured muscle with exogenous mitochondria by mitochondrial transplant therapy (MTT) would provide an improved environment and increased potential for repair after a pathological injury [80].

#### 3.9.1. Mitochondrial Regulation of the Extracellular Matrix in Muscle Regeneration

Previous studies have reported that the extracellular matrix (ECM), composed mainly of muscle collagen and other non-contractile tissue, may have an essential role in regulating MSC-induced muscle regeneration [450,451]. However, the importance of mitochondria availability to MSCs or fibroblasts to moderate MSC-directed muscle ECM repair is unknown. Our previous work suggests that loss of SIRT1—which is an important regulator of mitochondrial biogenesis via activation of PGC1α [452]—in MSCs reduces the restoration of muscle function after a pathological injury [59]. In addition, reducing mitochondrial biogenesis and Drp1 expression increases fibrosis [453,454]. In contrast, reducing mitochondria-induced ROS accumulation (in part as a result of impaired or inadequate mitophagy) can lower muscle fibrosis [455]. Since reducing PGC1α-regulated mitochondrial biogenesis during MSC differentiation can inhibit muscle repair, our recent study hypothesized that increasing mitochondrial abundance in injured muscle would reduce muscle fibrosis and improve the restoration of muscle function [80]. Our data show that increasing mitochondria in injured muscle reduces non-contractile tissue deposition during MSC-regulated muscle repair [80].

#### 3.9.2. Mitochondria Enhancement Improves Muscle Regeneration

**Figure 6:**
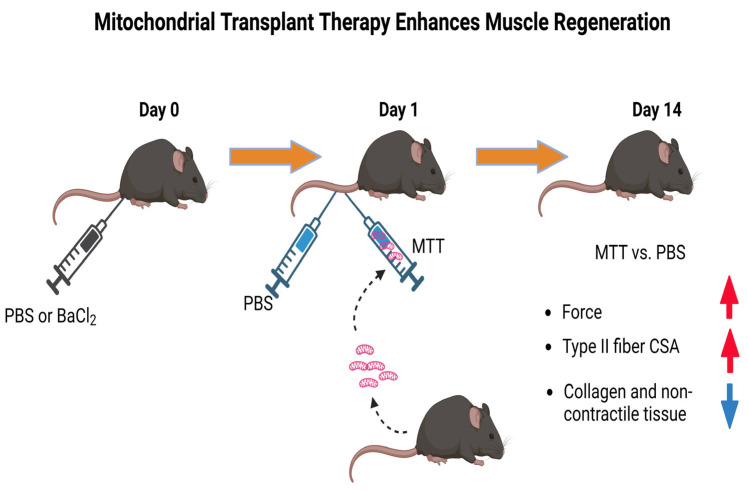
Mitochondrial Transplant Therapy (MTT). A hindlimb muscle of a mouse was injected with PBS as a control with the opposite limb injected with BaCl_2_ to induce muscle pathological injury. One day after the pathological injury, mice received either PBS as a sham treatment, or exogenous mitochondria that were isolated from tissue of a donor mouse (MTT, mitochondrial transplant therapy) through a tail vein. Fourteen days after the initial pathological injury, mice treated with MTT had greater total maximal force, larger type II fibers and less collagen and non-contractile tissue in the BaCl_2_ injured muscles as compared to the uninjured intra-animal control muscle.

Multiple studies have shown that mitochondrial transplantation therapy (MTT), which provides healthy donor mitochondria into ischemic myocardium [456,457,458,459], neural tissue [460], or skeletal muscle [461], can improve recovery from ischemia-reperfusion pathological injury. Donor mitochondria that are harvested from another tissue source can be incorporated into mesenchymal stem cells to improve arterial lung, neural [460], and cardiac tissue repair [462,463]. We have recently reported that the addition of healthy donor mitochondria to injured skeletal muscle can improve the restoration of neuromuscular function. In our study we report the novel finding that the systemic delivery of mitochondria can enhance muscle regeneration and restore muscle function following BaCl_2_-induced pathological injury in the muscles of mice (Figure 6). MTT appeared to enhance the regeneration of mouse muscle preferentially in the type IIB fibers, which is the population of fibers that normally have the lowest percentage of mitochondria. However, MTT did not improve the initial period of regeneration, and it only provided a beneficial effect in the repair period of 7 to 14 days after the initial pathological injury [80]. This is a period that would have occurred after the initial inflammation and MSC proliferation periods and which corresponds to the period of MSC differentiation and potentially mitochondrial biogenesis along with enhanced mitophagy. This, however, is consistent with the prior literature that indicates that increasing mitochondrial biogenesis/mitochondrial abundance during differentiation [464,465,466,467] results in improved muscle regeneration [80]. Thus, MTT provides an exogenous pool of healthy mitochondria that increases muscle differentiation and repair, and improves muscle function as compared to recovery from pathological injury without MTT [80]. This provides a promising therapeutic approach to manipulate mitochondrial involvement in muscle repair (Figure 6). The precise mechanism by which MTT improved muscle repair is currently unknown and is under investigation.

## 4. Conclusions

Mitochondria abundance and proteostasis have important roles in regulating MSC function in response to regeneration and repair after muscle physiological or pathophysiological injury. Initially, mitophagy and fission regulated fragmentation of mitochondria are important during the early phases of MSC proliferation and renewal of the stem cell population. In part, this is needed to clear mitochondria that are damaged as a result of the initial pathological injury and the acute inflammation response to the injury. The early responses of repair and MSC expansion also parallel the increases in Pax7 acetylation. After a sufficient pool of MSCs are available, the cells undergo differentiation. This is driven by Pax7 deacetylation and an elevation in mitochondrial biogenesis and mitochondrial fusion. However, mitophagy is also important during differentiation to remove damaged mitochondria and ensure a healthy mitochondrial pool. Increasing the mitochondrial content of injured muscle during the MSC differentiation phase by MTT improves the rate of muscle regeneration and reduces fibrosis and non-muscle proteins in response to muscle repair. Future studies are needed to determine what mechanisms drive improved regeneration after pathological or physiological injury in response to MTT. It is possible that the additional mitochondrial content in regenerating muscle could contribute to increasing total energy production for protein assembly. Alternatively, MTT could trigger increased mitophagy or contribute mitochondrial DNA to host mitochondria. Furthermore, we have observed that many mitochondria are perinuclear in location following MTT, and therefore it is possible that this location provides an optimal position for calcium buffering and limiting nucleoplasmic calcium loading in skeletal muscle, as is the case in cardiac muscle cells [51]. In addition, other anabolic signaling pathways may be enhanced through MTT. Although the mechanism(s) that regulate MTT-regulated enhancement of regeneration of injured muscle are not known, together, the findings to date suggest that mitochondria have multiple layers of regulatory responses that modulate muscle regeneration after a pathological or physiological injury, and that increasing the mitochondrial content of injured muscle increases its rate of repair.

## Figures and Tables

**Table 1:** Mitochondrial signaling in inflammation.

TLR9	Inflammation, muscle atrophy, increase cytokines (IL-6)	[54,175,176,180,181,182,183]
MFN1/2 loss	Atrophy, inflammation	[54,176,177,178]
Parkin1 loss	Increase in cytokines (cytokines IL-6, IFNβ1, IL-12, IL-13, XXCL1, CCL2, CCL4)	[187]
Pink1 loss	Increase in immune genes, and cytokines IL-6, IFNβ1, TNFα, IL-1β CCL2, IL-12(p70), IL-13, IL-17, CXCL1, CCL4	[187,200,201]
cGAS-STING	Increase type I Interferon	[187,210,211,212,213,214]
Mitophagy/mitochondria stress	NRLP3, mtDAMPs (mtDNA, TFAM, cardiolipin)	[177,188,189,190,191]

**Table 2:** Altered expression of mitochondria mRNA, miRNA, and proteins after a pathological injury or during repair following pathological injury.

Condition	mRNA Increases	Protein Increases	Functional Increases	References
Eccentric exercise	PCG1α, TFAM	SDHa, CKmt2, ANT1	mitochondria biogenesis and function	[342]
Ischemic injury	TBC1D15	TBC1D15	mitochondrial homeostasis	[343]
CTX injury	Nmrk2		differentiation of myoblasts	[344]
Ischemic injury		GJA1-20k	mitochondrial size, recruits actin to mitochondria (decreased ROS)	[345]
Myogenesis/repair	PERM1, PGC1α		mitochondria biogenesis and mitochondrial respiratory function	[346,347,348]
Aging, denervation injury, ischemia	Bax, ATF6, GRP-78, caspase 3	Bax, caspase 3, caspase 9, cytochrome c	apoptosis and atrophy	[13,148,273,349]
CTX muscle repair		SIRT1, p53	mitochondria size, Complex III activity	[59]
CTX muscle repair		SIRT1, p53, SOD1, CAT	mitochondria size, Complex I, III, ATPase activity	[350]
CTX and freezing muscle repair	PGC1β, PRC, NRF-1, NRF-2, TFAM, ERR, Drp1		Mitochondrial fission, fusion and biogenesis	[351,352]
CTX muscle repair	Drp1, ULK1, BNIP3, and MAP1LC3-II		Mitochondrial fission, fusion, and biogenesis	[353,354]
Freezing muscle repair	Drp1 BNIP3, Pink1, Parkin		Mitochondrial fission, mitophagy	[88,355]

Activating transcription factor 6 (ATF6), Bcl-2 Associated X-protein (Bax), adenine nucleotide translocase type 1 (ANT1), Creatine kinase (CKm), Complex II succinate dehydrogenase complex flavoprotein subunit A (SDHa), Mitochondria creatine kinase (CKmt2), Citrate synthase (CS), Glucose regulated protein-78 (GRP78), Cyclophilin D, mitochondrial single-stranded DNA binding protein 1 (mtSSB), Estrogen-related receptor a (ERR), Dynamin 1-like (Drp1), BCL1 interacting protein (BNIP3), microtubule-associated proteins 1A/1B light chain 3B (MAP1LC3-II), Mitochondrial transcription factor A (TFAM), Mitochondrial calcium uniporter (MCU), Nicotinamide riboside kinase 2 (Nmrk2), Nuclear respiratory factor 1 (NRF-1), Nuclear respiratory factor 1 (NRF-2), Peroxisome proliferator-activated receptor-gamma coactivator-alpha (PGC1α), Peroxisome proliferator-activated receptor-gamma coactivator-beta (PGC1β), PGC1-related coactivator (PRC), PGC1 and estrogen-related receptor (ERR)-induced regulator, muscle 1 (PERM1), Sirtuin-1 (SIRT1), Superoxide dismutase (SOD), TBC1 domain family member 15 (TBC1D15), Unc-51-like kinase 1 (ULK1).

## Data Availability

Data available in a publicly accessible form that is associated with each of the respective papers cited in this article.
